# RALFL34 regulates formative cell divisions in Arabidopsis pericycle during lateral root initiation

**DOI:** 10.1093/jxb/erw281

**Published:** 2016-07-18

**Authors:** Evan Murphy, Lam Dai Vu, Lisa Van den Broeck, Zhefeng Lin, Priya Ramakrishna, Brigitte van de Cotte, Allison Gaudinier, Tatsuaki Goh, Daniel Slane, Tom Beeckman, Dirk Inzé, Siobhan M. Brady, Hidehiro Fukaki, Ive De Smet

**Affiliations:** ^1^Division of Plant and Crop Sciences, School of Biosciences, University of Nottingham, Loughborough LE12 5RD, UK; ^2^Department of Plant Systems Biology, VIB, B-9052 Ghent, Belgium; ^3^Department of Plant Biotechnology and Bioinformatics, Ghent University, B-9052 Ghent, Belgium; ^4^Department of Medical Protein Research, VIB, 9000 Ghent, Belgium; ^5^Department of Biochemistry, Ghent University, 9000 Ghent, Belgium; ^6^Department of Plant Biology and Genome Center, University of California Davis, One Shields Avenue, Davis, CA 95616, USA; ^7^Department of Biology, Graduate School of Science, Kobe University, Kobe 657-8501, Japan; ^8^Department of Cell Biology, Max Planck Institute for Developmental Biology, D- 72076 Tübingen, Germany; ^9^Centre for Plant Integrative Biology, University of Nottingham, Loughborough LE12 5RD, UK

**Keywords:** *Arabidopsis thaliana*, ERF, GATA23, lateral root initiation, RAPID ALKALINIZATION FACTOR (RALF).

## Abstract

In plants, many signalling molecules, such as phytohormones, miRNAs, transcription factors, and small signalling peptides, drive growth and development. However, very few small signalling peptides have been shown to be necessary for lateral root development. Here, we describe the role of the peptide RALFL34 during early events in lateral root development, and demonstrate its specific importance in orchestrating formative cell divisions in the pericycle. Our results further suggest that this small signalling peptide acts on the transcriptional cascade leading to a new lateral root upstream of GATA23, an important player in lateral root formation. In addition, we describe a role for ETHYLENE RESPONSE FACTORs (ERFs) in regulating *RALFL34* expression. Taken together, we put forward RALFL34 as a new, important player in lateral root initiation.

## Highlight

We describe the role of RALFL34 during early events in lateral root development, and demonstrate its specific importance in orchestrating formative cell divisions in the pericycle.

## Introduction

Root plasticity is one of the main adaptive traits enabling plants to cope with an ever-changing environment. Formation and positioning of lateral roots along the longitudinal primary root axis plays a vital role in nutrient acquisition and water uptake. Lateral roots are formed post-embryonically from the pericycle cells adjacent to the xylem poles (Malamy and Benfey, 1997; Dubrovsky *et al.*, 2001; De Smet *et al.*, 2006). Their initiation and development occur in a regular way, and depend largely on the plant hormone auxin (Laskowski *et al.*, 1995; Malamy and Benfey, 1997; Beeckman *et al.*, 2001; Casimiro *et al.*, 2001; Lavenus *et al.*, 2013). The development of lateral root primordia goes through several well-described stages, with the first stages being essential for proper lateral root primordium development (Malamy and Benfey, 1997; De Smet *et al.*, 2008, 2010; Lucas *et al.*, 2013; von Wangenheim *et al.*, 2016). Typically, stage 1 comprises two rounds of asymmetric cell divisions of a small set of pericycle founder cells, forming smaller daughter cells with distinct cell fates. At stage 2, a rotation in the plane of division occurs; the cells divide periclinally toward the outer tissues forming an outer layer and an inner layer. This division normally occurs first in the two most central cells, followed by the adjacent cells. The most peripheral cells do not divide periclinally, so, as the central cells expand radially, the establishment of the lateral root primordia dome shape materializes. In stages 3–7, numerous rounds of anticlinal and periclinal cell divisions occur, establishing distinct tissue layers eventually mimicking the organization of the primary root tip. Stage 8 involves few cell divisions; however, rapid cell expansion results in penetration of the overlying tissue, emerging from the primary root. Several of the underlying genes and proteins that are involved in priming, founder cell specification or activation, and initiating and advancing lateral root development have been identified through transcript profiling and the use of gain-of-function and loss-of-function mutants, such as SOLITARY ROOT (SLR)/IAA14, AUXIN RESPONSE FACTOR7 (ARF7), ARF19, LATERAL ORGAN BOUNDARIES-DOMAIN16 (LBD16), LBD29, GATA23, ARABIDOPSIS CRINKLY4 (ACR4), and several others (Fukaki *et al.*, 2002; Tatematsu *et al.*, 2004; Okushima *et al.*, 2005, 2007; De Smet *et al.*, 2008; De Rybel *et al.*, 2010; Lavenus *et al.*, 2013). Less information, however, is known on cellular communication during lateral root development, specifically, through the relatively recently discovered small signalling peptides.

Small signalling peptides have been shown to play a wide variety of roles in the plant, with recent evidence also showing their involvement in lateral root development (Murphy *et al.*, 2012; Czyzewicz *et al.*, 2013; Delay *et al.*, 2013*b*; Tavormina *et al.*, 2015). For example, CLE-LIKE (CLEL)/GOLVEN (GLV)/ROOT GROWTH FACTOR (RGF) peptides inhibit pericycle cell divisions when overexpressed (Matsuzaki *et al.*, 2010; Meng *et al.*, 2012; Whitford *et al.*, 2012; Fernandez *et al.*, 2013, 2015). INFLORESCENCE DEFICIENT IN ABSCISSION (IDA) and its receptors HAESA (HAE) and HAESA-LIKE2 (HSL2) play a role in lateral root emergence (Kumpf *et al.*, 2013). Various C-TERMINALLY ENCODED PEPTIDEs (CEPs) reduce (emerged) lateral root density when the synthetic peptide is exogenously applied or endogenously overexpressed (Delay *et al.*, 2013*a*; Roberts *et al.*, 2016). CLAVATA3/EMBRYO SURROUNDING REGION (CLE) peptides inhibit lateral root emergence when overexpressed (Araya *et al.*, 2014) and also regulate lateral root development through BIN2-mediated phosphorylation of ARFs (Cho *et al.*, 2014). A recently discovered peptide, AUXIN-RESPONSIVE ENDOGENOUS POLYPEPTIDE 1 (AREP1), promotes lateral root organogenesis in the presence of auxin (Yang *et al.*, 2014). Finally, RAPID ALKALINIZATION FACTOR (RALF) peptides in Arabidopsis regulate various processes predominantly through regulating cell expansion (Pearce *et al.*, 2001; Srivastava *et al.*, 2009; Mingossi *et al.*, 2010; Atkinson *et al.*, 2013; Bergonci *et al.*, 2014; Morato do Canto *et al.*, 2014). Some RALF peptides, such as RALF1, RALF19, and RALF23, increase emerged lateral root densities as demonstrated by *RALF*-silenced transgenic lines and decrease densities as shown by the use of *RALF*-overexpressing lines (Atkinson *et al.*, 2013; Bergonci *et al.*, 2014). Taken together, it is peculiar that the majority of the so far characterized small signalling peptides have a negative impact on root architecture. Speculatively, this might imply that these small signalling peptides act as specific, negative regulators of, for example, the dominant, promoting effect of auxin.

Here, we describe the role of RALF-LIKE 34 (RALFL34) in lateral root initiation, and position this small signalling peptide in the transcriptional cascade leading to a new lateral root. In addition, we describe a role for ETHYLENE RESPONSE FACTORs (ERFs) in regulating *RALFL34* expression.

## Materials and methods

### Plant materials

We used the following lines in our research: Columbia-0 (Col-0), Landsberg *erecta* (L*er*), *ralfl34-1* (SALK_004441) (Alonso *et al.*, 2003), *ralfl34-2* (JIC_SGT4223) (Parinov *et al.*, 1999), *pGATA23::NLS:GFP* (De Rybel *et al.*, 2010), WAVE131Y (Geldner *et al.*, 2009), *pRALFL34*_*869bp*_ *::n3xRFP* (see below), and *p35S::ERF9-GR* (see below).

### Growth of *Arabidopsis thaliana* seedlings

Seeds were surface sterilized (70% ethanol for 2min, 10% bleach for 15min, dH_2_O for five washes) and then stratified at 4 °C for 2 d. Seeds were then plated on half-strength Murashige and Skoog (1/2 MS) agar plates (2.154g l^–1^ MS, 0.1g l^–1^ myo-inositol, 0.5g l^–^ MES, 10g l^–^ bacteriological agar, pH 5.7 with 1M KOH) and germinated vertically under constant white light at 21 °C.

### Genotyping of T-DNA insertion lines

The following T-DNA insertion mutant lines were used and genotyped in our investigations: *ralfl34-1* (SALK_004441) using the following genotyping primers, FW primer TGACTAACCAAAAA GTCCACG; REV primer, ACGGGACCTCTAGCTCTGAAG; and T-DNA primer, ATTTTGCCGATTTCGGAAC; and *ralfl34-2* (JIC_SGT4223) using the following genotyping primers, FWprimer, ATGGCAGCTTCGTCTCTC; REV primer, CTATCTCCGGCATCGAGT; and T-DNA primer, ACGGTCGGGAAACTAGCTCTAC.

### Constructs

The *pRALFL34::LUC* was generated as follows: the 400bp *RALFL34* promoter fragment was PCR amplified using the *RALFL34* forward (CAACTGGACCCATCCGAA) and reverse primer (CGGCGATTGTTGGGGGA) and cloned into the pGem-T-easy vector. After sequencing confirmation, the 416bp *RAFL34* promoter fragment was released from the pGem^®^-T Easy vector with restriction enzymes *Pst*I and *Nco*I and then cloned into the LucTrap vector (Calderon-Villalobos *et al.*, 2006; Lau *et al.*, 2011; De Smet *et al.*, 2013). The sequence of the final construct was confirmed. *ERF9* and *ERF4* were cloned into *pJIT60* as follows: the *ERF9* and *ERF4* coding sequences were PCR amplified using *ERF9* (ATGGCTCCAAGACAGGCG and CTAAACGTCCACCACCGGT) and *ERF4* primers (ATGGCCAAGATGGGCTTG and TCAGGCCTGTTCCGA TGG), and were cloned into the pGem^®^-T-Easy vector. The sequences of the plasmids containing either *ERF9* or *ERF4* were confirmed, and the *ERF9* and *ERF4* coding sequences were released by the restriction enzyme *Eco*RI and cloned into the pJIT60 vector (Schwechheimer *et al.*, 1998; Lau *et al.*, 2011; De Smet *et al.*, 2013). The sequences of the final constructs were confirmed. For the yeast one-hybid (Y1H) experiments, *pRALFL34* fragments were cloned as follows: 416bp and 869bp *RALFL34* promoter fragments were PCR amplified using *RALFL34* forward (CAACTGGACCCATCCGAA or CAGTATCAGGCTTGTGTTCA) and reverse primer (CGGCGATT GTTGGGGGA or GGCGATTGTTGGGGGAAA) and then cloned into the entry vector pENTR™ 5'-TOPO (Invitrogen). The entry clones containing the 416bp or 869bp *RALFL34* promoters were then recombined to yield *promoter::HIS3* and *promoter::LacZ* reporter constructs (Deplancke *et al.*, 2004; Brady *et al.*, 2011) and their sequences confirmed.

### Y1H

Y1H assays were performed as previously described (Gaudinier *et al.*, 2011). Interactions were called for transcription factors that activated at least one reporter assay.

### Transgenic lines

We generated *pRALFL34::n3xRFP*, using a previously published vector backbone (Slane *et al.*, 2014) and a *RALFL34* promoter fragment of 869bp (counting from ATG). To generate the *p35S::ERF9-GR* line, the coding sequence of ERF9 without a STOP codon, a glucocorticoid domain (GR), and the constitutive 35S promoter were cloned in pDONR221, pDONRP2RP3, and pDONRP4P1R, respectively, with Gateway Cloning^®^. A multisite LR recombination combined the entry vectors into the destination vector pK8m34GW-FAST. The sequences of both entry vectors and the expression vector were confirmed. Both *pRALFL34::n3xRFP* and *p35S::ERF9-GR* constructs were transformed into *Agrobacterium* sp. and then floral dipped into Col-0 plants (Clough and Bent, 1998).

### Induction of lateral roots

Lateral root induction was performed through mechanical (Ditengou *et al.*, 2008) or gravitropic bending (Péret *et al.*, 2012; Lavenus *et al.*, 2015; Voβ *et al.*, 2015), as previously described. Quantification of *pGATA23::NLS:GFP* fluorescence was analysed on Col-0 (wild type) and *ralfl34-1* seedlings. Total fluorescing nuclei (in all tissues) were counted from the quiescent centre (QC) moving up the root for ~2500 μm and density measurements were calculated as ‘fluorescing nuclei in *n* μm’.

### DEX treatment

For dexamethasone (DEX) treatments, seedlings were grown *in vitro* on 1/2 MS plates (7.5g l^–^ agar) overlaid with a nylon mesh (Prosep, 20 µm pore size). At 7 d after sowing, the mesh with seedlings was transferred to plates with 1/2 MS medium (7.5g l^–^ agar) and plates with 1/2 MS medium containing 5 µM DEX (Sigma).

### Microscopic analysis

Phenotyping was analysed under a Leica stereo dissection microscope at varying magnifications to observe and count the emerged lateral roots. Lateral roots were counted, marked, and then photographed. Root lengths were measured from the bottom of the hypocotyl to the root tip, using ImageJ software (http://imagej.nih.gov/ij/). Lateral root staging of the *ralfl34-1* and *ralfl34-2* mutants was performed on a Leica DMRB microsystem using differential interference contrast (DIC). Fluorescent seedlings were analysed on a Nikon confocal microscope utilizing both an Argon 488 laser and a 514 HeNe laser at ×20 and ×40 magnification.

### RALFL34 auxin response quantification

Arabidopsis (ecotype Col-0) seeds were surface sterilized, stratified at 4 °C for 2 d, then plated on 1/2 MS medium and grown vertically under constant white light at 21 °C for 4 d post-germination. Seedlings were transferred to 1/2 MS liquid medium on the fourth day to acclimate overnight. Seedlings were transferred to liquid 1/2 MS containing 1-naphthaleneacetic acid (NAA) at a concentration of 10 μM (or ethanol control) and were grown for a further 0, 1, 2, 4, 6, 8, 12, and 24h; at each time point, the roots of 10 seedlings were excised, frozen in liquid nitrogen, and then total RNA extracted as below.

### Quantitative real-time PCR (qRT-PCR)

Total RNA was extracted from lines of interest using an RNeasy kit (Qiagen) as per the manufacturer’s instructions. cDNA was generated from 1 µg or 200ng of total RNA via oligo(dT) primers using iScript (BioRad) or Superscript II (Invitrogen), respectively, as per the instructions. Quantitative PCRs were performed in at least two technical repeats, using the SYBR Green QPCR Master Mix (Quanta Biosciences), in our modified reaction protocol: Master mix, 7 μl per reaction [forward primer (100 μM), 0.1 μl per reaction; reverse primer (100 μM), 0.1 μl per reaction; SYBR (2×), 6 μl per reaction; dH_2_O, 0.8 μl per reaction] and template cDNA (diluted 1/100 in dH_2_O, 5 μl per reaction. Alternatively, the LightCycler^®^ 480 SYBR Green I Master (2×) in Light Cycler 480 (Roche) was used, plates were filled with the JANUS Automated Workstation Perkin Elmer, primers were used at 0.5 µM, and cDNA was diluted 8× (total volume=5 µl=0.5 µl of template, 2 µl of primers, 2.5 µl; 2× mix). Gene-specific quantification was performed with intron-spanning primer pairs (where possible) designed using Primer3 software (http://bioinfo.ut.ee/primer3/). Expression was normalized using *AT5G60390* (CTGGAGGTTTTGAGGCTGGTAT and CCAAGGGTGAAAGCAAGAAGA) and/or *AT3G 60830* (ACTCTTCCTGATGGACAGGTG and CTCAACGATT CCATGCTCCT) and/or *AT4G16100* (GGAGATTAAGC AACCTGAGGAGTG and GTGGTGGTGGTGGAGGA GAC). Primers used for qPCR: *RALFL34* (CGATGCCGG AGATAGATGAAG or TGGCAGCTTCGTCTCTCAA and CAATCCACCCCTCACGACT or TGGAGAGAAATGAAA GTGAGAAGAG), *GATA23* (AGTGAGAATGAAAGAAGA GAAGGG or GGGGACAATTAGGTGTTGCA and GTGGCT GCGAATAATATGAATACC or CTCCTTCGTTTCTTCA CCGC), *ERF4* (TTTCTCGAGCTGAGTGACCA and GTCGGAGGAGAAGCACAGTC), and *ERF9* (AGAGAGTTT CGTGGCTCCAA and CCACCGTCGTTAACCGTAGT). All qRT-PCR experiments were performed in three biological replicates (unless otherwise specified) and 2–3 technical repeats, and the data presented represent means ±SE.

### *In silico* analyses

CORNET analyses were done using standard settings in the TF tool on https://bioinformatics.psb.ugent.be/cornet/versions/cornet3.0/main/tf (De Bodt *et al.*, 2010, 2012; De Bodt and Inzé, 2013). We used SignalP 4.1 (Petersen *et al.*, 2011) with standard settings to predict the presence and location of a signal peptide cleavage site in RALFL34. Genevestigator (Zimmermann *et al.*, 2004, 2005; Hruz *et al.*, 2008) was probed using the standard application. For the identification of transcription factor-binding sites, we used CIS-BP (Weirauch *et al.*, 2014), and to search for the identified motif matrices in promoter sequences we used Regulatory Sequence Analysis Tools (RSAT) (Medina-Rivera *et al.*, 2015). We used Arabidopsis eFP Browser (Winter *et al.*, 2007) absolute or relative values to gain insight into expression patterns of selected genes.

### Transient activity assays

BY-2 protoplast assays were performed as previously described (Vanden Bossche *et al.*, 2013), using reporter (*LucTrap*, encoding firefly luciferase) and effector constructs (*pJIT60*).

## Results and Discussion

### RALFL34 regulates pericycle cell division patterns during lateral root initiation

**Fig. 1.**
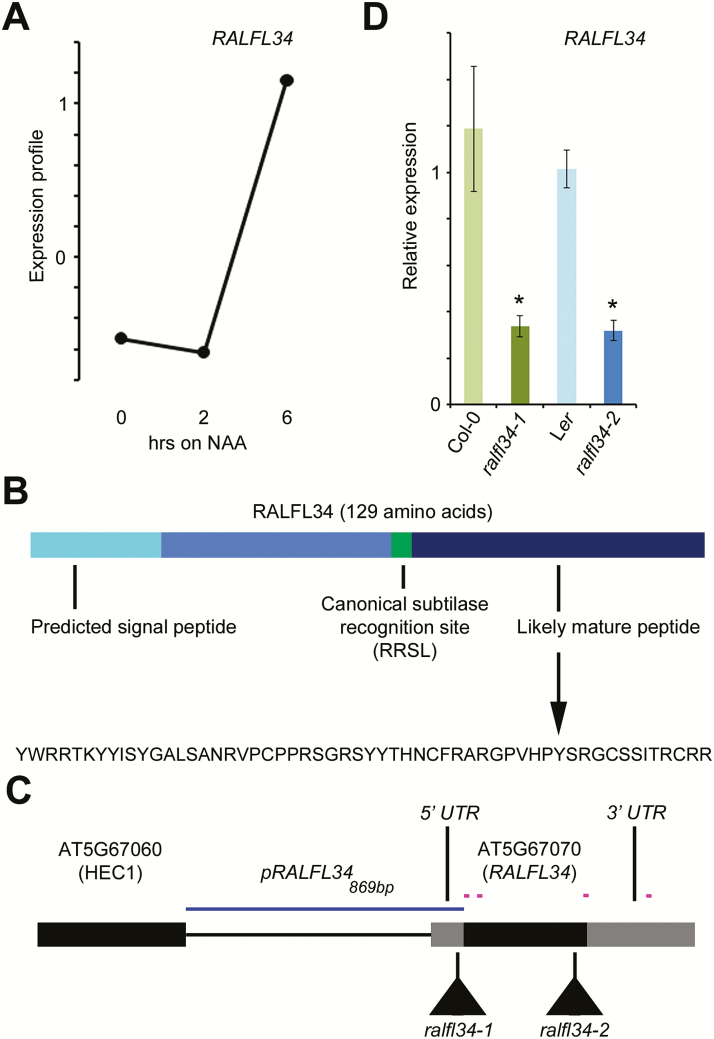
*RALFL34* expression and gene/protein topology. (A) Expression profile of *RALFL34* in the pericycle of seedlings subjected to lateral root inducible system [after mixed model analysis; data from De Smet *et al.*, (2008)]. Seedlings were grown on NPA (1-*N*-naphthylphthalamic acid; 0h) for 3 d. NAA, 1-naphthaleneacetic acid. (B) Schematic of RALFL34 protein, highlighting the putative signal peptide and likely mature peptide region and sequence. (C) Schematic of the *RALFL34* gene, with indication of qPCR primers (pink lines), position of T-DNA insertion, and promoterregion (blue line). (D) *RALFL34* expression as monitored through qPCR in *ralfl34-1* and *ralfl34-2* roots at 5 d after germination and their respective control lines, Col-0 and L*er*. The graph depicts the average of three biological repeats (and 3–6 technical repeats) ±SE. Student’s *t*-test with *P*-value <0.01.

In a transcriptome analysis focusing on early stages of lateral root initiation and utilizing a lateral root inducible system based on auxin treatment leading to synchronous induction of lateral roots (Himanen *et al.*, 2002, 2004; De Rybel *et al.*, 2010), *RALFL34* (*AT5G67070*) expression was up-regulated, together with a small set of 14 potential key regulatory genes for asymmetric cell division and/or cell fate specification during lateral root initiation, including *ARABIDOPSIS CRINKLY4* (*ACR4*) (De Smet *et al.*, 2008) (Fig. 1A). *RALFL34* encodes a member of a family of cysteine-rich small signalling peptides (Murphy and De Smet, 2014) and probably gives rise to a 56 amino acid mature peptide (Fig. 1B). While members of the RALF family have been functionally characterized (Pearce *et al.*, 2001; Olsen *et al.*, 2002; Haruta *et al.*, 2008, 2014; Matos *et al.*, 2008; Srivastava *et al.*, 2009; Atkinson *et al.*, 2013; Bergonci *et al.*, 2014; Morato do Canto *et al.*, 2014; Li *et al.*, 2015), no data are so far available for RALFs in the context of lateral root initiation.

**Fig. 2.**
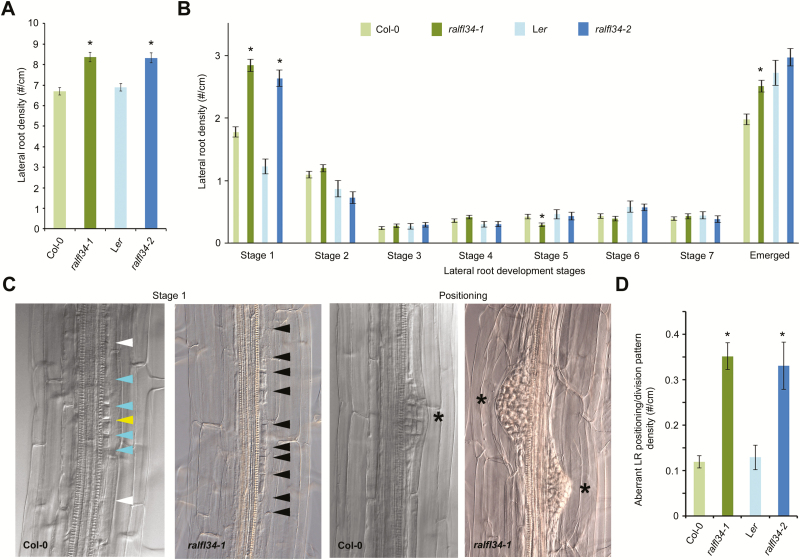
Lateral root phenotypes in *ralfl34* mutants. (A, B) Lateral root density in *ralfl34-1* (*n*=69) and *ralfl34-2* (*n*=29) compared with their respective controls, Col-0 (*n*=79) and L*er* (*n*=20) depicted as total lateral root density, including all stages (A) and split into lateral root stages 1–7 and emerged lateral roots (according to Malamy and Benfey, 1997) (B) at 7 d after germination. Stage 1 includes regions with extra pericycle cell divisions. (C, D) Representative DIC pictures (C) and quantification (D) of aberrant stage 1, additional pericycle divisions, or unusually positioned lateral roots in *ralfl34-1* (*n*=69) and *ralfl34-2* (*n*=29) compared with their respective controls, Col-0 (*n*=79) and L*er* (*n*=20). An asterisk indicates a lateral root primordium. Arrowheads separate pericycle cells: central (yellow) and outer position (white) for adjacent pericycle cells undergoing two rounds of asymmetric cell divisions (blue) and extra rounds of divisions (black). All graphs show the average ±SE of the indicated sample numbers. *P<0.05 according to Student’s *t*-test compared with control.

To validate a role for RALFL34 in lateral root initiation, we identified two T-DNA lines, namely *ralfl34-1* (in Col-0) and *ralfl34-2* (in L*er*), with significantly reduced *RALFL34* expression (Fig. 1C,D). Both *ralfl34-1* and *ralfl34-2* were analysed with respect to overall lateral root density, lateral root stage distribution, and pericycle division patterns. Overall, *ralfl34-1* and *ralfl34-2* displayed, respectively, a 25% and 21% increase in total lateral root density (emerged and non-emerged) compared with their control line (Fig. 2A; Supplementary Fig. S1 at *JXB* online). A more detailed analysis quantifying the different stages of lateral root development in *ralfl34-1* and *ralfl34-2* revealed that this is probably due to an enrichment of stage 1 lateral root primordia [which also included regions with divisions that did not fully resemble a typical stage 1 primordium (as shown in Fig. 2D)] compared with the control (Fig. 2B). Furthermore, we observed a 3-fold increase in ‘aberrant’ pericycle division patterns (extra cell divisions flanking the primordia) or unusually positioned (defined as lateral root primordia being closer to each other than 400 µm) in *ralfl34-1* and *ralfl34-2* compared with their control line (Fig. 2C, D; Supplementary Fig. S1). Based on these observations, we concluded that RALFL34 plays a role during lateral root initiation and probably acts to restrict (proliferative) cell divisions and/or mediate the spatial distribution of asymmetric founder cell divisions in the pericycle along the root’s longitudinal axis.

### RALFL34 is expressed before asymmetric pericycle cell division

**Fig. 3.**
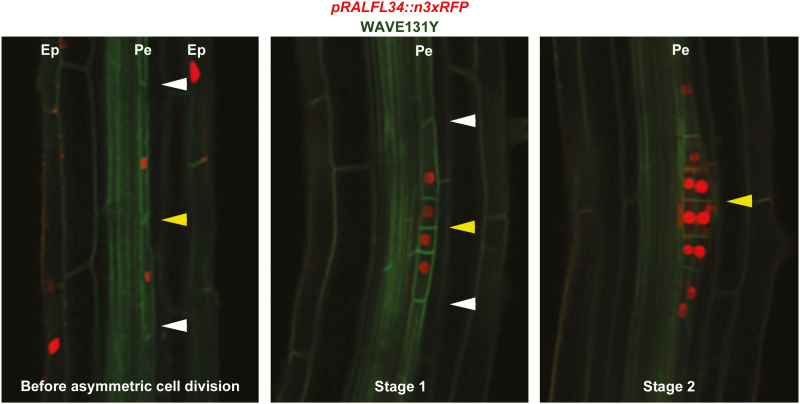
*RALFL34* expression during lateral root initiation as visualized through *pRALFL34::n3xRFP* (red) and WAVE 131Y (green). Arrowheads separate pericycle cells: central (yellow) and outer position (white) for adjacent pericycle cells undergoing two rounds of asymmetric cell divisions. Lateral root development stages are indicated. Ep, epidermis. Pe, pericycle (at xylem pole).

**Fig. 4.**
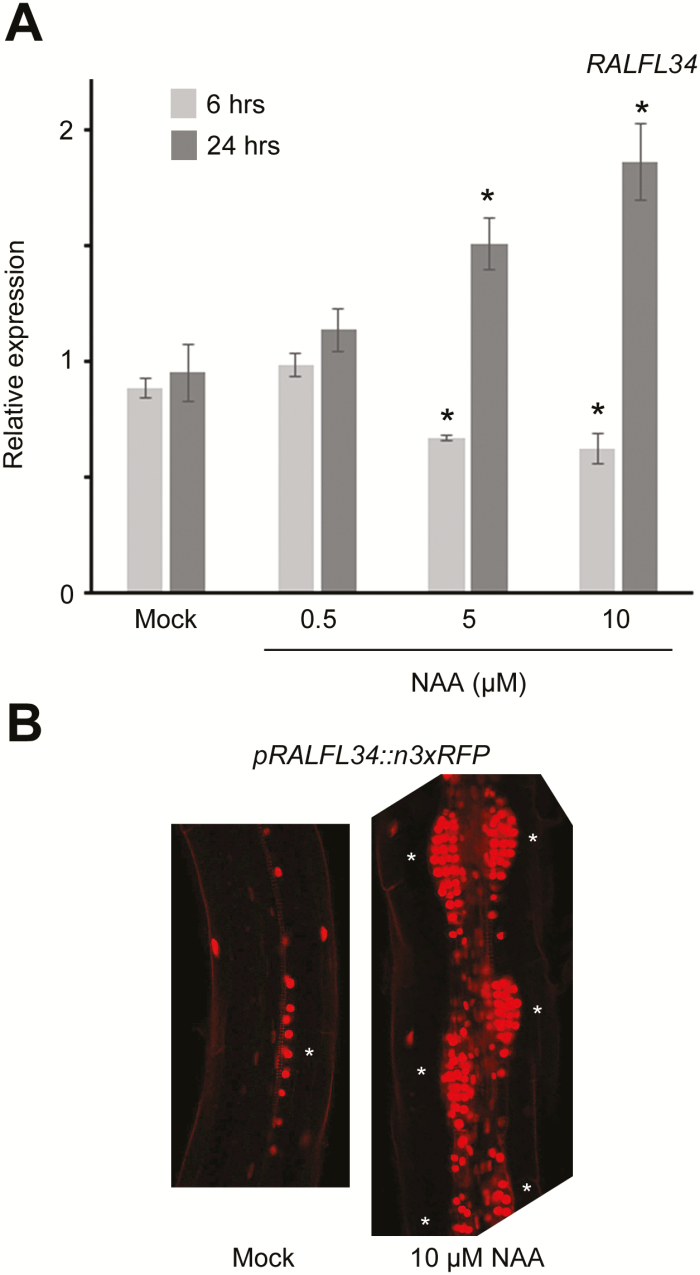
Auxin-mediated regulation of *RALFL34* expression. (A) Expression of *RALFL34* after 6h and 24h treatment with NAA. The graph shows the average ±SE of three biological repeats. *P<0.05 according to Student’s *t*-test compared with mock. (B) Representative images of *pRALFL34::n3xRFP* (red) expression upon 24h of NAA treatment. An asterisk indicates the position of lateral root initiation/primordium and/or dividing pericycle.

To investigate at which stage during lateral root development *RALFL34* is expressed, we generated a *pRALFL34*_*869bp*_ *::n3xRFP* line (referred to as *pRALFL34::n3xRFP*) and combined this with a yellow fluorescent protein-NPSN12 plasma membrane marker line (WAVE131Y) (Geldner *et al.*, 2009). *RALFL34* is expressed in xylem pole pericycle cells before any visible sign of asymmetric cell division (Fig. 3A). At a later stage, *RALFL34* is strongly expressed in the small central cells of the lateral root initiation site, but also in the larger flanking cells (Fig. 3B, C). In addition, we detected *RALFL34* expression in the epidermis (Fig. 3A). Given that lateral root development is controlled by auxin at various levels (Lavenus *et al.*, 2013), we explored if *RALFL34* expression is regulated by auxin in the root. Treatment with the synthetic auxin NAA (1-naphthaleneacetic acid) showed minor, but significant, concentration-dependent down-regulation after 6h and minor, but significant, concentration-dependent transcriptional up-regulation following a 24h treatment (Fig. 4A). Primary auxin-responsive genes are generally significantly differentially regulated within 2h after exposure to auxin (Abel *et al.*, 1994; Oeller and Theologis, 1995). This suggests that *RALFL34* is probably not a (positively regulated) primary auxin response gene, but acts nevertheless downstream of an auxin response module. It should be noted that these results are obtained from auxin-treated seedlings grown on control medium before treatment, while the initial identification of *RALFL34* (see above; Fig. 1A) was based on auxin treatment synchronously inducing asymmetric cell divisions in the pericycle following growth on the auxin transport inhibitor NPA. Furthermore, this change in *RALFL34* expression is likely to be due to initiation of multiple formative asymmetric divisions, as an increased number of lateral root primordia induced by the NAA treatment are observed (Fig. 4B). Interestingly, *RALFL34* was recently identified as having a mobile mRNA in a root to shoot direction (Thieme *et al.*, 2015), suggesting that the *RALFL34* domain—also in the root—is likely to be broader than reported by our *pRALFL34::n3xRFP* line. In this context, Arabidopsis eFP Browser data suggested the presence of *RALFL34* mRNA in above-ground plant parts (Supplementary Fig. S2), but this can be derived from mobile mRNA and/or local expression.

### ‘Flanking’ *RALFL34* expression is associated with lateral root initiation

**Fig. 5.**
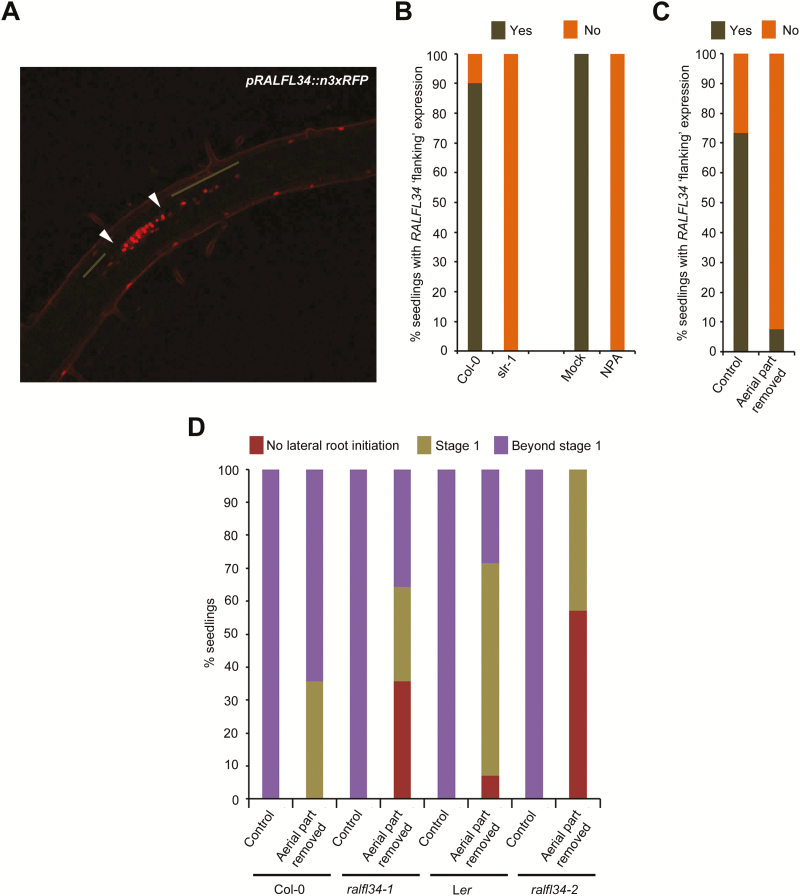
*RALFL34* ‘flanking’ expression. (A) Representative image of *pRALFL34::n3xRFP* expression with expression in the lateral root primordium (between white arrowheads) and flanking the lateral root primordium (brown line) upon bending. (B, C) Percentage of seedlings showing *pRALFL34::n3xRFP* ‘flanking’ expression in (B) the wild type (Col-0) versus *slr* and mock (DMSO) versus NPA-treated seedlings upon bending and in (C) seedlings with aerial tissues removed. (D) Percentage of seedlings, with aerial tissues removed, which failed to initiate lateral roots (red), developed a stage 1 primordium (khaki), or progressed beyond stage 1 (purple), 20h post-mechanical bending.

Interestingly, the *pRALFL34::n3xRFP* expression around the lateral root initiation site often showed a ‘flanking’ expression profile, which appears to be contained within the pericycle, that extended from the lateral root initiation site shootward and/or rootward (25/30 lateral root primordia) (Fig. 5A). We established that this ‘flanking’ expression is indeed associated with the formation of a lateral root. For this, we subjected seedlings that do not display lateral root initiation, namely grown on NPA or in the *slr-1* background, to mechanical root tip bending to induce a lateral root, as was previously described (Ditengou *et al.*, 2008). Under these experimental conditions (and 20h after the bending), we did not observe any lateral root initiation-associated (and thus probably no lateral root formation) or ‘flanking’ *RALFL34* expression in the pericycle (Fig. 5B). Since RALFL34 seems to affect the number of lateral roots along the longitudinal primary root axis, we speculated that this ‘flanking’ expression might play a role in regularly positioning these lateral roots.

### A shoot-derived signal affects RALFL34 expression around the lateral root primordium

Since shoot-derived signals are involved in various steps of lateral root development (Reed *et al.*, 1998; Bhalerao *et al.*, 2002; McAdam *et al.*, 2016), we explored the involvement of the shoot (as a source of auxin or another signal) in establishing the ‘flanking’ *pRALFL34::n3xRFP* expression in the root. Therefore, we removed the aerial tissues (hypocotyl and upward) and subjected *pRALFL34::n3xRFP* plants to the gravitropic bending assay (Péret *et al.*, 2012; Lavenus *et al.*, 2015; Voβ *et al.*, 2015) to induce the formation of a lateral root. *pRALFL34::n3xRFP* expression was assessed for 20h after the removal of their aerial tissues and following mechanical root tip bending. Seedlings grown under control conditions (no aerial tissues removed) showed >70% *pRALFL34::n3xRFP* flanking expression after 20h gravistimulation (Fig. 5C). In contrast, seedlings with aerial tissues removed had only 10% with *pRALFL34::n3xRFP* flanking expression (Fig. 5C). These data strongly suggest that a shoot-derived signal is required for *pRALFL34::n3xRFP* ‘flanking’ expression.

To observe precisely the impact of removing shoot-derived signals on RALFL34-mediated lateral root formation, we utilized the gravitropic bending assay in combination with aerial tissue (hypocotyl and upward) removal on *ralfl34* mutants. Without removing aerial tissues, there was no significant difference between any of the controls or mutant lines 20h post-bending, as all lateral roots progressed beyond stage 1 (Fig. 5D). However, 20h post-bending and having removed the aerial tissues, we observed a reduction in the percentage of seedlings with lateral root initiation events in *ralfl34-1* (64%) and *ralfl34-2* (43%), compared with the Col-0 (100%) and L*er* (93%) control, respectively (Fig. 5D). In addition, we also observed a reduction in the percentage of lateral root primordia that progressed beyond stage 1 in *ralfl34-1* (56%) and *ralfl34-2* (0%), compared with the Col-0 (64%) and L*er* (31%) control, respectively (Fig. 5D). Taken together, our results suggest that RALFL34 is required for lateral root initiation (and progression beyond this stage) and appears to interpret a shoot-derived signal that is possibly not auxin. In addition, these results and the *RALFL34* ‘flanking’ expression pattern might indicate that RALFL34 is part of a lateral inhibition mechanism based on positive and negative feedback.

### *RALFL34* expression is negatively regulated by ERFs

**Fig. 6.**
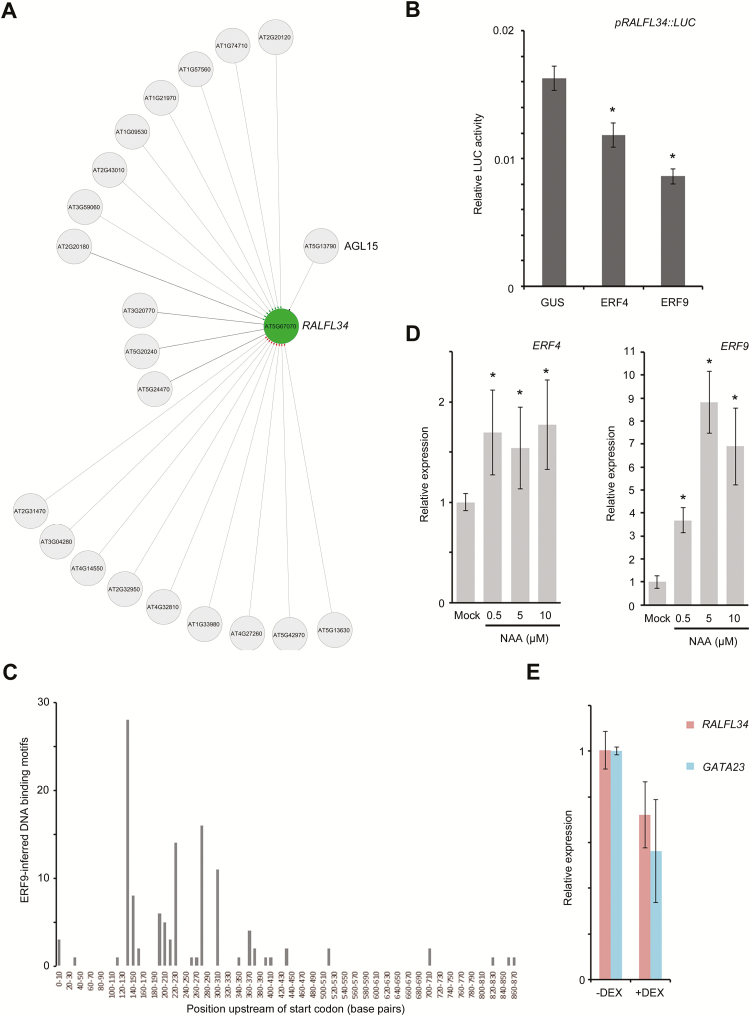
ERFs regulated *RALFL34* expression. (A) *In silico* generated transcription factor network, highlighting putatively interacting proteins with *RALFL34* in CORNET. Interactions are as follows: confirmed (full line), unconfirmed (dotted line), indirect (diamond), direct+unknown (disc), direct+activation (arrowhead), direct+repression [line activation (green), repression (red), and unknown (black)]. (B) Luciferase (LUC) activity upon co-expression of pRALFL34::LUC and GUS (control) or ERF4/9 in protoplasts. Luciferase assay was performed three times, each time with eight biological replicates. The graph shows the average of all 24 data points ±SE. Student’s *t*-test with *P*-value <0.01. (C) Number of ERF9-inferred DNA-binding motifs according to their position upstream of the coding sequence (start codon=position 0). (D) *ERF4* and *ERF9* expression upon 6h of NAA treatment at the indicated concentrations. The graph shows the average ±SE error of three biological repeats. *P<0.05 according to Student’s *t*-test compared with mock. (E) Expression of *RALFL34* and *GATA23* in an inducible ERF9 overexpression line (*35S::ERF9-GR*) after 4h of DEX-induced overexpression of *ERF9*.

To gain insight into the regulation of *RALFL34* expression (and possibly the shoot-derived signal), we initially performed an *in silico* analysis using CORNET, which allows the integration of regulatory interaction data sets accessible through the transcription factor tool (De Bodt *et al.*, 2010, 2012; De Bodt and Inzé, 2013). This revealed a set of 21 transcription factors potentially regulating *RALFL34* expression (Fig. 6A), of which one was also differentially expressed in the pericycle during lateral root initiation (De Smet *et al.*, 2008) and/or all those that could be checked were to some extent differentially expressed in the Arabidopsis Lateral Root initiation eFP Browser (Supplementary Table S1). Next, we performed an enhanced yeast one-hybrid (eY1H) analysis (Gaudinier *et al.*, 2011) on two *RALFL34* upstream regulatory regions of different length (869bp or 416bp; with the original idea to narrow down the regulatory region arbitrarily), with a collection of transcription factors expressed in the root stele which includes pericycle cells (Supplementary Table S1). This revealed a set of 34 transcription factors potentially regulating *RALFL34* expression, of which four were also differentially expressed in the pericycle during lateral root initiation (De Smet *et al.*, 2008) and/or all were to some extent differentially expressed in the Arabidopsis Lateral Root initiation eFP Browser (Supplementary Table S1). This list of interacting transcription factors showed limited overlap with the CORNET data, namely only AGAMOUS-LIKE 15 (AGL15) (Supplementary Table 1). AGL15 has already been characterized extensively (Perry *et al.*, 1999; Fernandez *et al.*, 2000, 2014; Wang *et al.*, 2002, 2004; Harding *et al.*, 2003; Tang and Perry, 2003; Adamczyk *et al.*, 2007; Hill *et al.*, 2008; Zheng *et al.*, 2009; Patharkar and Walker, 2015), and *RALFL34* was also listed in a genome-wide identification of *in vivo* AGL15-binding sites (Zheng *et al.*, 2009). To determine if AGL15 is sufficient to regulate *RALFL34* expression *in planta*, we performed experiments using a heterologous *in vivo* protoplast system which monitors gene expression quantitatively by activation of a luciferase (LUC) reporter (Lau *et al.*, 2011; Vanden Bossche *et al.*, 2013). Based on the results (data not shown), we concluded that although AGL15 can physically bind the promoter, it was not able to reproducibly regulate the expression of *pRALFL34*_*416bp*_ *::LUC* in this transient system. We therefore decided to focus on members of the ERF protein family, as this family was represented by seven out of 34 Y1H hits (Supplementary Table 1). Next, we specifically tested an interaction between *RALFL34* expression and (the arbitrarily selected) ERF4 and ERF9 in protoplasts. This revealed a significant down-regulation of *LUC* expression (Fig. 6B), which is in agreement with ERF4 and ERF9 containing a repression domain (Licausi *et al.*, 2013). In addition, we did an *in silico* search for ERF4 and ERF9 DNA-binding motifs. ERFs are known to bind directly to the *cis*-element called a GCC-box containing the core 5′-GCCGCC-3′ sequence (Ohme-Takagi and Shinshi, 1995). We could indeed find ERF4-binding motifs and ERF9 inferred binding motifs in the promoter of *RALFL34*. Interestingly, 92% of the identified motifs were found in the first 400bp of the promoter (Fig. 6C). We further confirmed this interaction *in planta* using a transgenic *ERF9-GR* line. This revealed that in the roots, ERF9 could indeed (mildly) down-regulate *RALFL34* expression (Fig. 6E). The ERF-mediated down-regulation of *RALFL34* expression possibly links *RALFL34* expression to a regulation by ethylene. However, an analysis of available, general Arabidopsis Genevestigator and eFP Browser data did not reveal any significant differential regulation of *RALFL34* expression upon ACC (1-aminocyclopropane-1-carboxylic acid) treatment (data not shown). However, to conclude this convincingly, a more detailed and cell- or tissue-specific expression profiling would be needed to evaluate the influence of ACC on *RALFL34* expression. Interestingly, *ERF4* and mainly *ERF9* expression is up-regulated by auxin in the root (Fig. 6D). In addition, based on eFP Browser data, *ERF4* and *ERF9* appear to be ethylene inducible (Supplementary Fig. S3). In future, we will further look into this potential hormone crosstalk, but it remains possible that this ERF4– or ERF9−*RALFL34* network edge is not associated with ethylene and/or lateral root development, as *RALFL34* is more broadly expressed in the plant (Supplementary Fig. S2).

### RALFL34 acts upstream of GATA23

**Fig. 7.**
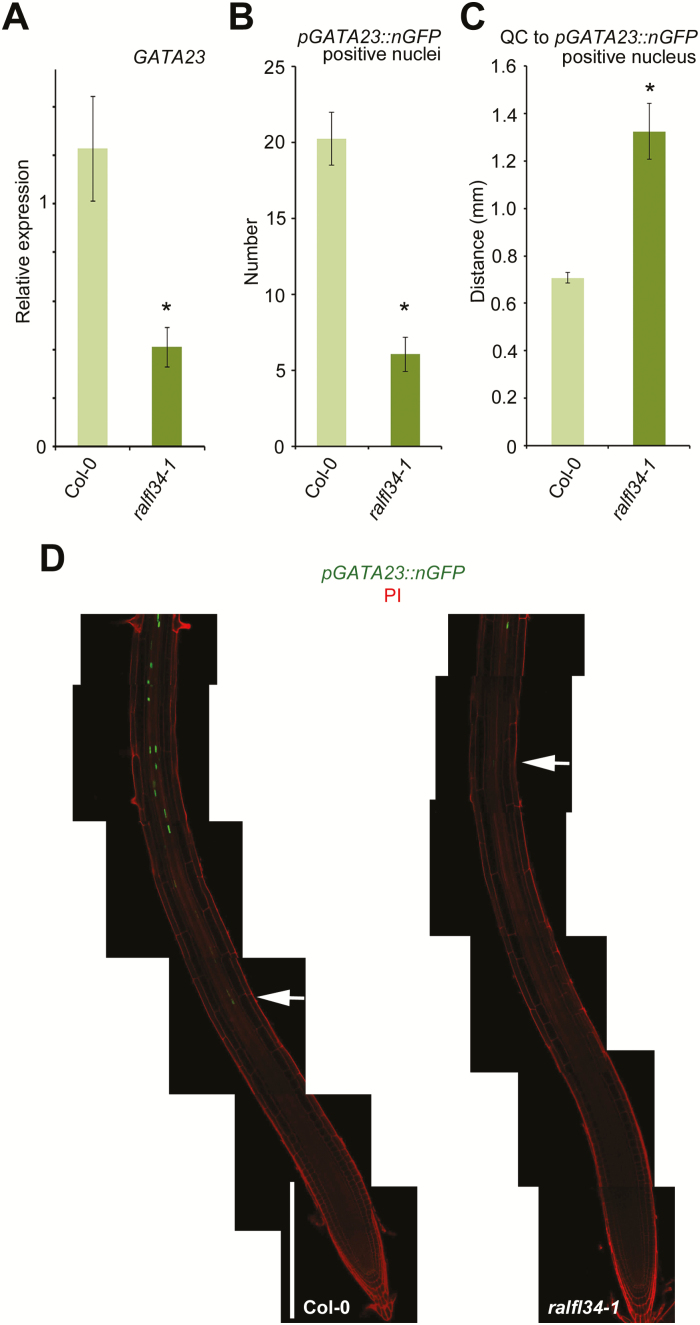
*GATA23* expression downstream of RALFL34. (A) *GATA23* expression in wild-type (Col-0) and *ralfl34-1* seedling roots 5 d after germination as monitored through qPCR. The graph depicts the average of three biological repeats (and 3–6 technical repeats) ±SE. Student’s *t*-test with *P*-value <0.01. (B) Representative images of *pGATA23::nGFP* expression in the wild type (Col-0) and *ralfl34-1*, white arrows indicating the first GFP expressing nucleus. White bar=300 μm. (C, D) Average number of *pGATA23::nGFP*-positive nuclei (C) and average length from the quiescent centre (QC) to the first nGFP-positive nucleus in the root tips of Col-0 and *ralfl34-1* (D). Graphs show the average ±SE of 30 seedlings. *P<0.05 according to Student’s *t*-test compared with the wild type.

Finally, we aimed to position RALFL34 with respect to known regulators in the cascade that leads to lateral root development (Atkinson *et al.*, 2014; Behringer and Schwechheimer, 2015; Vermeer and Geldner, 2015). One of the earliest markers for lateral root initiation is GATA23, a transcription factor that controls founder cell identity (De Rybel *et al.*, 2010), and we therefore analysed *GATA23* expression in *ralfl34-1* roots. qRT-PCR analyses on seedling roots revealed a significant down-regulation of *GATA23* expression levels in *ralfl34-1* compared with the control (Fig. 7A). To confirm this, we evaluated the *pGATA23::NLS:GFP* line (De Rybel *et al.*, 2010) in the *ralfl34-1* background. This revealed overall less green fluorescent protein (GFP)-positive nuclei in the root (Fig. 7B, C). In addition, the location of the first GFP-positive nucleus, with respect to the QC, was higher up the root (Fig. 7D). Based on these results, we conclude that RALFL34 probably acts upstream and as a positive regulator of *GATA23*. However, the lateral root initiation phenotypes of *ralfl34-1* (this work) and a *GATA23* ^*RNAi*^ line (fewer emerged lateral roots and fewer stage 1/2 lateral root primordia) (De Rybel *et al.*, 2010) do not appear to match. There are potentially additional regulators of GATA23 or possibly altered expression of *GATA23* in *ralfl34-1* is an indirect effect, for example through a perturbed auxin balance, especially in view of *GATA23* being a highly auxin-responsive gene (Supplementary Fig. S4) (De Rybel *et al.*, 2010). Interestingly though, *GATA23* expression seems also to be down-regulated upon DEX-induced ERF9 activity, suggesting that RALF34 might represent a component connecting ERF9 signalling with downstream transcriptional regulation of *GATA23* (an indirect effect caused by the direct down-regulation of *RALFL34*) or, alternatively, ERF9 acts on *GATA23* in a parallel pathway (a direct effect by the binding of ERF9 to the promoter of *GATA23*) (Fig. 6E).

### Conclusion

**Fig. 8.**
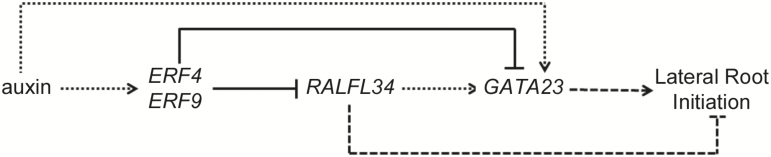
Schematic representation of possible RALFL34-mediated regulation of lateral root initiation and regulation of *ERF4*, *ERF9*, *RALFL34*, and *GATA23* expression in the root. Dotted lines (possibly/likely) indirect effects; dashed lines, developmental impact; full line, (likely) direct effect.

In this work, we used a candidate gene approach [based on differential expression in our transcriptomics data (De Smet *et al.*, 2008)] to identify molecular components of lateral root initiation and associated asymmetric cell division. Our results indicate a strong developmental role for the small signalling peptide RALFL34 during the early stages of lateral root development. *RALFL34* expression is down-regulated by auxin (Fig. 8). Interestingly, upstream of *RALFL34* expression are auxin-inducible and ethylene-inducible ERFs that down-regulate *RALFL34* expression (Fig. 8). Previously, ethylene has been shown to affect the ability of pericycle cells to undergo lateral root initiation, probably through interfering with auxin accumulation (Ivanchenko *et al.*, 2008; Negi et al., 2008, 2010; Lewis *et al.*, 2011). With respect to a shoot-derived signal, our data confirm that this is required for the normal progression of lateral root development from stage 1, and which acts downstream of primary auxin responsiveness (Bhalerao *et al.*, 2002; Marchant *et al.*, 2002; Ditengou *et al.*, 2008). In addition, it appears that RALFL34 is interpreting a shoot-derived signal to drive the progression from founder cell to stage 1 primordia. Concurrently, *RALFL34* expression in the flanking pericycle cells induced by a shoot-derived signal might be essential to restrain cell proliferation in the neighbouring pericycle cells because *ralfl34* mutants are characterized by extra cell divisions in these regions. One possibility is that this shoot-derived signal is auxin, as indeed this hormone plays a role in regulating lateral root development (Lavenus *et al.*, 2013). However, given that the primary effect of auxin is repression of *RALFL34* expression, we believe other signals are likely to be involved. For example, in view of lateral root positioning, a carotenoid-derived molecule was already shown to play a role (Van Norman *et al.*, 2014). In addition, it was recently shown that *ERF109* expression is strongly up-regulated by methyl jasmonate (MeJA), and that ERF109 regulates lateral root development in response to MeJA (Cai *et al.*, 2014). In this context, however, it is interesting to note that in the leaves *ERF9* is transcriptionally down-regulated following short-term MeJA and salicylic acid (SA) treatment (Maruyama *et al.*, 2013). Finally, we have shown that RALFL34 acts genetically upstream of GATA23 (Fig. 8), but it is not clear if this is a direct regulation or indirect due to perturbed auxin accumulation and/or responsiveness. However, *GATA23* expression seems also to be regulated downstream of ERF9 (Fig. 8). Taken together, our data revealed an as yet unreported role for RALF peptides in lateral root initiation, and position RALFL34 as a possible earlier marker for founder cell identity than GATA23. In future, the spatio-temporal regulation of and genetic interactions within this small network will need to be investigated in detail in the root in order to establish its biological role further. In addition, what the underlying mechanism and targets of RALFL34 action are remain to be explored. In this respect, RALF peptides have been shown to be linked to Ca^2+^ release (Haruta *et al.*, 2008, 2014; Morato do Canto *et al.*, 2014), which—in turn—is associated with mechanical stress (Richter *et al.*, 2009). Interestingly, mechanical stimulation of roots causes increases in Ca^2+^ in epidermal, cortical, endodermal, and pericycle cells of roots (potentially through stretch-activated Ca^2+^ channels) (Richter *et al.*, 2009). Furthermore, lateral root initiation also requires root bending, which can be seen as a mechanical stimulus (Laskowski *et al.*, 2008; Kircher and Schopfer, 2016; Scheres and Laskowski, 2016). In future, it will be important to determine to what extent Ca^2+^ levels are perturbed in *ralfl34* roots under normal conditions and during (mechanical) root bending compared with wild-type roots. Another explanation might come from the recently identified RALF1 receptor, namely FERONIA (FER), a member of the highly expressed malectin receptor-like kinase family (Haruta *et al.*, 2014). It was shown that RALF1 binds to FER, causing downstream phosphorylation on a plasma membrane-associated H^+^-ATPase (AHA2), resulting in an increased apoplastic pH and a decrease in cell wall elongation. AHA2 (*aha2*) has since been shown also to cause a decrease in lateral root density, correlating with a downstream mechanism as to why *35S::RALF1* lines have decreased lateral root density (Mlodzinska *et al.*, 2015). Overall, RALFL34 possibly affects ion balance in the pericycle (and/or surrounding tissues) to regulate cell divisions associated with lateral root initiation.

## Supplementary data

Supplementary data are available at *JXB* online.

Figure S1. Lateral root phenotypes in *ralfl34-1* and *ralfl34-2.*

Figure S2. Absolute expression value for *RALFL34* in above-ground organs.

Figure S3. Relative expression values for *ERF4* and *ERF9* upon ACC treatment.

Figure S4. *GATA23* expression upon NAA treatment.

Table S1. CORNET and Y1H data.

Supplementary Data

## References

[CIT0001] Abel S Oeller PW Theologis A 1994 Early auxin-induced genes encode short-lived nuclear proteins. Proceedings of the National Academy of Sciences, USA 91, 326–330. 10.1073/pnas.91.1.326 PMC42940 8278386

[CIT0002] Adamczyk BJ Lehti-Shiu MD Fernandez DE 2007 The MADS domain factors AGL15 and AGL18 act redundantly as repressors of the floral transition in Arabidopsis. The Plant Journal 50, 1007–1019. 17521410 10.1111/j.1365-313X.2007.03105.x

[CIT0003] Alonso JM Stepanova AN Leisse TJ 2003 Genome-wide insertional mutagenesis of Arabidopsis thaliana. Science 301, 653–657. 12893945 10.1126/science.1086391

[CIT0004] Araya T Miyamoto M Wibowo J 2014 CLE–CLAVATA1 peptide–receptor signaling module regulates the expansion of plant root systems in a nitrogen-dependent manner. Proceedings of the National Academy of Sciences, USA 11, 2029–2034. 10.1073/pnas.1319953111 PMC3918772 24449877

[CIT0005] Atkinson JA Rasmussen A Traini R Voβ U Sturrock C Mooney SJ Wells DM Bennett MJ 2014 Branching out in roots: uncovering form, function, and regulation. Plant Physiology 166, 538–550. 25136060 10.1104/pp.114.245423 PMC4213086

[CIT0006] Atkinson NJ Lilley CJ Urwin PE 2013 Identification of genes involved in the response of Arabidopsis to simultaneous biotic and abiotic stresses. Plant Physiology 162, 2028–2041. 23800991 10.1104/pp.113.222372 PMC3729780

[CIT0007] Beeckman T Burssens S Inzé D 2001 The peri-cell-cycle in Arabidopsis. Journal of Experimental Botany 52, 403–411. 11326046 10.1093/jexbot/52.suppl_1.403

[CIT0008] Behringer C Schwechheimer C 2015 B-GATA transcription factors—insights into their structure, regulation, and role in plant development. Frontiers in Plant Science 6, 90. 25755661 10.3389/fpls.2015.00090 PMC4337238

[CIT0009] Bergonci T Ribeiro B Ceciliato PH Guerrero-Abad JC Silva-Filho MC Moura DS 2014 Arabidopsis thaliana RALF1 opposes brassinosteroid effects on root cell elongation and lateral root formation. Journal of Experimental Botany 65, 2219–2230. 24620000 10.1093/jxb/eru099 PMC3991750

[CIT0010] Bhalerao RP Eklof J Ljung K Marchant A Bennett M Sandberg G 2002 Shoot-derived auxin is essential for early lateral root emergence in Arabidopsis seedlings. The Plant Journal 29, 325–332. 11844109 10.1046/j.0960-7412.2001.01217.x

[CIT0011] Brady SM Zhang L Megraw M 2011 A stele-enriched gene regulatory network in the Arabidopsis root. Molecular Systems Biology 7, 459. 21245844 10.1038/msb.2010.114 PMC3049412

[CIT0012] Cai XT Xu P Zhao PX Liu R Yu LH Xiang CB 2014 Arabidopsis ERF109 mediates cross-talk between jasmonic acid and auxin biosynthesis during lateral root formation. Nature Communications 5, 5833. 10.1038/ncomms6833 25524530

[CIT0013] Calderon-Villalobos LI Kuhnle C Li H Rosso M Weisshaar B Schwechheimer C 2006 LucTrap vectors are tools to generate luciferase fusions for the quantification of transcript and protein abundance in vivo. Plant Physiology 141, 3–14. 16684932 10.1104/pp.106.078097 PMC1459313

[CIT0014] Casimiro I Marchant A Bhalerao RP 2001 Auxin transport promotes Arabidopsis lateral root initiation. The Plant Cell 13, 843–852. 11283340 10.1105/tpc.13.4.843 PMC135543

[CIT0015] Cho H Ryu H Rho S 2014 A secreted peptide acts on BIN2-mediated phosphorylation of ARFs to potentiate auxin response during lateral root development. Nature Cell Biology 16, 66–76. 24362628 10.1038/ncb2893

[CIT0016] Clough SJ Bent AF 1998 Floral dip: a simplified method for Agrobacterium-mediated transformation of Arabidopsis thaliana. The Plant Journal 16, 735–743. 10069079 10.1046/j.1365-313x.1998.00343.x

[CIT0017] Czyzewicz N Yue K Beeckman T Smet ID 2013 Message in a bottle: small signalling peptide outputs during growth and development. Journal of Experimental Botan*y* 64, 5281–5296. 24014870 10.1093/jxb/ert283

[CIT0018] De Bodt S Carvajal D Hollunder J Van den Cruyce J Movahedi S Inzé D 2010 CORNET: a user-friendly tool for data mining and integration. Plant Physiology 152, 1167–1179. 20053712 10.1104/pp.109.147215 PMC2832254

[CIT0019] De Bodt S Hollunder J Nelissen H Meulemeester N Inzé D 2012 CORNET 2.0: integrating plant coexpression, protein–protein interactions, regulatory interactions, gene associations and functional annotations. New Phytologist 195, 707–720. 22651224 10.1111/j.1469-8137.2012.04184.x

[CIT0020] De Bodt S Inzé D 2013 A guide to CORNET for the construction of coexpression and protein–protein interaction networks. Methods in Molecular Biology 1011, 327–343. 23616008 10.1007/978-1-62703-414-2_26

[CIT0021] Delay C Imin N Djordjevic MA 2013 *a* CEP genes regulate root and shoot development in response to environmental cues and are specific to seed plants. Journal of Experimental Botany 64, 5383–5394. 24179096 10.1093/jxb/ert332

[CIT0022] Delay C Imin N Djordjevic MA 2013 *b* Regulation of Arabidopsis root development by small signaling peptides. Frontiers in Plant Science 4, 352. 24046775 10.3389/fpls.2013.00352 PMC3764427

[CIT0023] Deplancke B Dupuy D Vidal M Walhout AJ 2004 A gateway-compatible yeast one-hybrid system. Genome Research 14, 2093–2101. 15489331 10.1101/gr.2445504 PMC528925

[CIT0024] De Rybel B Vassileva V Parizot B 2010 A novel aux/IAA28 signaling cascade activates GATA23-dependent specification of lateral root founder cell identity. Current Biology 20, 1697–1706. 20888232 10.1016/j.cub.2010.09.007

[CIT0025] De Smet I Lau S Ehrismann JS Axiotis I Kolb M Kientz M Weijers D Jurgens G 2013 Transcriptional repression of BODENLOS by HD-ZIP transcription factor HB5 in Arabidopsis thaliana. Journal of Experimental Botany 64, 3009–3019. 23682118 10.1093/jxb/ert137 PMC3697942

[CIT0026] De Smet I Lau S Voβ U 2010 Bimodular auxin response controls organogenesis in Arabidopsis. Proceedings of the National Academy of Sciences, USA 107, 2705–2710. 10.1073/pnas.0915001107 PMC2823897 20133796

[CIT0027] De Smet I Vanneste S Inzé D Beeckman T 2006 Lateral root initiation or the birth of a new meristem. Plant Molecular Biology 60, 871–887. 16724258 10.1007/s11103-005-4547-2

[CIT0028] De Smet I Vassileva V De Rybel B 2008 Receptor-like kinase ACR4 restricts formative cell divisions in the Arabidopsis root. Science 322, 594–597. 18948541 10.1126/science.1160158

[CIT0029] Ditengou FA Teale WD Kochersperger P 2008 Mechanical induction of lateral root initiation in Arabidopsis thaliana. Proceedings of the National Academy of Sciences, USA 105, 18818–18823. 10.1073/pnas.0807814105 PMC2596224 19033199

[CIT0030] Dubrovsky JG Rost TL Colon-Carmona A Doerner P 2001 Early primordium morphogenesis during lateral root initiation in Arabidopsis thaliana. Planta 214, 30–36. 11762168 10.1007/s004250100598

[CIT0031] Fernandez A Drozdzecki A Hoogewijs K Vassileva V Madder A Beeckman T Hilson P 2015 The GLV6/RGF8/CLEL2 peptide regulates early pericycle divisions during lateral root initiation. Journal of Experimental Botany 66, 5245–5256. 26163695 10.1093/jxb/erv329 PMC4526922

[CIT0032] Fernandez A Hilson P Beeckman T 2013 GOLVEN peptides as important regulatory signalling molecules of plant development. Journal of Experimental Botany 64, 5263–5268. 23975768 10.1093/jxb/ert248

[CIT0033] Fernandez DE Heck GR Perry SE Patterson SE Bleecker AB Fang SC 2000 The embryo MADS domain factor AGL15 acts postembryonically. Inhibition of perianth senescence and abscission via constitutive expression. The Plant Cell 12, 183–198. 10662856 10.1105/tpc.12.2.183 PMC139757

[CIT0034] Fernandez DE Wang CT Zheng Y Adamczyk BJ Singhal R, Hall PK Perry SE 2014 The MADS-domain factors AGAMOUS- LIKE15 and AGAMOUS-LIKE18, along with SHORT VEGETATIVE PHASE and AGAMOUS-LIKE24, are necessary to block floral gene expression during the vegetative phase. Plant Physiology 165, 1591–1603. 24948837 10.1104/pp.114.242990 PMC4119041

[CIT0035] Fukaki H Tameda S Masuda H Tasaka M 2002 Lateral root formation is blocked by a gain-of-function mutation in the SOLITARY-ROOT/IAA14 gene of Arabidopsis. The Plant Journal 29, 153–168. 11862947 10.1046/j.0960-7412.2001.01201.x

[CIT0036] Gaudinier A Zhang L Reece-Hoyes JS 2011 Enhanced Y1H assays for Arabidopsis. Nature Methods 8, 1053–1055. 22037706 10.1038/nmeth.1750 PMC3821074

[CIT0037] Geldner N Denervaud-Tendon V Hyman DL Mayer U Stierhof YD Chory J 2009 Rapid, combinatorial analysis of membrane compartments in intact plants with a multicolor marker set. The Plant Journal 59, 169–178. 19309456 10.1111/j.1365-313X.2009.03851.x PMC4854200

[CIT0038] Harding EW Tang W Nichols KW Fernandez DE Perry SE 2003 Expression and maintenance of embryogenic potential is enhanced through constitutive expression of AGAMOUS-Like 15. Plant Physiology 133, 653–663. 14512519 10.1104/pp.103.023499 PMC219041

[CIT0039] Haruta M Monshausen G Gilroy S Sussman MR 2008 A cytoplasmic Ca2+ functional assay for identifying and purifying endogenous cell signaling peptides in Arabidopsis seedlings: identification of AtRALF1 peptide. Biochemistry 47, 6311–6321. 18494498 10.1021/bi8001488

[CIT0040] Haruta M Sabat G Stecker K Minkoff BB Sussman MR 2014 A peptide hormone and its receptor protein kinase regulate plant cell expansion. Science 343, 408–411. 24458638 10.1126/science.1244454 PMC4672726

[CIT0041] Hill K Wang H Perry SE 2008 A transcriptional repression motif in the MADS factor AGL15 is involved in recruitment of histone deacetylase complex components. The Plant Journal 53, 172–185. 17999645 10.1111/j.1365-313X.2007.03336.x

[CIT0042] Himanen K Boucheron E Vanneste S de Almeida Engler J Inzé D Beeckman T 2002 Auxin-mediated cell cycle activation during early lateral root initiation. The Plant Cell 14, 2339–2351. 12368490 10.1105/tpc.004960 PMC151221

[CIT0043] Himanen K Vuylsteke M Vanneste S 2004 Transcript profiling of early lateral root initiation. Proceedings of the National Academy of Sciences, USA 101, 5146–5151. 10.1073/pnas.0308702101 PMC387388 15051881

[CIT0044] Hruz T Laule O Szabo G Wessendorp F Bleuler S Oertle L Widmayer P Gruissem W Zimmermann P 2008 Genevestigator v3: a reference expression database for the meta-analysis of transcriptomes. Advances in Bioinformatics 2008, 420747. 19956698 10.1155/2008/420747 PMC2777001

[CIT0045] Ivanchenko MG Muday GK Dubrovsky JG 2008 Ethylene–auxin interactions regulate lateral root initiation and emergence in Arabidopsis thaliana. The Plant Journal 55, 335–347. 18435826 10.1111/j.1365-313X.2008.03528.x

[CIT0046] Kircher S Schopfer P 2016 Priming and positioning of lateral roots in Arabidopsis. An approach for an integrating concept. Journal of Experimental Botany 67, 1411–1420. 26712828 10.1093/jxb/erv541 PMC4762386

[CIT0047] Kumpf RP Shi CL Larrieu A Sto IM Butenko MA Peret B Riiser ES Bennett MJ Aalen RB 2013 Floral organ abscission peptide IDA and its HAE/HSL2 receptors control cell separation during lateral root emergence. Proceedings of the National Academy of Sciences, USA 110, 5235–5240. 10.1073/pnas.1210835110 PMC3612645 23479623

[CIT0048] Laskowski M Grieneisen VA Hofhuis H Hove CA Hogeweg P Maree AF Scheres B 2008 Root system architecture from coupling cell shape to auxin transport. PLoS Biology 6, e307. 19090618 10.1371/journal.pbio.0060307 PMC2602721

[CIT0049] Laskowski MJ Williams ME Nusbaum HC Sussex IM 1995 Formation of lateral root meristems is a two-stage process. Development 121, 3303–3310. 7588064 10.1242/dev.121.10.3303

[CIT0050] Lau S De Smet I Kolb M Meinhardt H Jurgens G 2011 Auxin triggers a genetic switch. Nature Cell Biology 13, 611–615. 21478855 10.1038/ncb2212

[CIT0051] Lavenus J Goh T Guyomarc’h S 2015 Inference of the Arabidopsis lateral root gene regulatory network suggests a bifurcation mechanism that defines primordia flanking and central zones. The Plant Cell 27, 1368–1388. 25944102 10.1105/tpc.114.132993 PMC4456640

[CIT0052] Lavenus J Goh T Roberts I 2013 Lateral root development in Arabidopsis: fifty shades of auxin. Trends in Plant Science 18, 450–458. 23701908 10.1016/j.tplants.2013.04.006

[CIT0053] Lewis DR Negi S Sukumar P Muday GK 2011 Ethylene inhibits lateral root development, increases IAA transport and expression of PIN3 and PIN7 auxin efflux carriers. Development 138, 3485–3495. 21771812 10.1242/dev.065102

[CIT0054] Li C Yeh FL Cheung AY 2015 Glycosylphosphatidylinositol-anchored proteins as chaperones and co-receptors for FERONIA receptor kinase signaling in Arabidopsis. Elife 4. 10.7554/eLife.06587 PMC4458842 26052747

[CIT0055] Licausi F Ohme-Takagi M Perata P 2013 APETALA2/Ethylene Responsive Factor (AP2/ERF) transcription factors: mediators of stress responses and developmental programs. New Phytologist 199, 639–649. 24010138 10.1111/nph.12291

[CIT0056] Lucas M Kenobi K von Wangenheim D 2013 Lateral root morphogenesis is dependent on the mechanical properties of the overlaying tissues. Proceedings of the National Academy of Sciences, USA 110, 5229–5234. 10.1073/pnas.1210807110 PMC3612681 23479644

[CIT0057] Malamy JE Benfey PN 1997 Organization and cell differentiation in lateral roots of Arabidopsis thaliana. Development 124, 33–44. 9006065 10.1242/dev.124.1.33

[CIT0058] Marchant A Bhalerao R Casimiro I Eklof J Casero PJ Bennett M Sandberg G 2002 AUX1 promotes lateral root formation by facilitating indole-3-acetic acid distribution between sink and source tissues in the Arabidopsis seedling. The Plant Cell 14, 589–597. 11910006 10.1105/tpc.010354 PMC150581

[CIT0059] Maruyama Y Yamoto N Suzuki Y Chiba Y Yamazaki K Sato T Yamaguchi J 2013 The Arabidopsis transcriptional repressor ERF9 participates in resistance against necrotrophic fungi. Plant Science 213, 79–87. 24157210 10.1016/j.plantsci.2013.08.008

[CIT0060] Matos JL Fiori CS Silva-Filho MC Moura DS 2008 A conserved dibasic site is essential for correct processing of the peptide hormone AtRALF1 in Arabidopsis thaliana. FEBS Letters 582, 3343–3347. 18775699 10.1016/j.febslet.2008.08.025

[CIT0061] Matsuzaki Y Ogawa-Ohnishi M Mori A Matsubayashi Y 2010 Secreted peptide signals required for maintenance of root stem cell niche in Arabidopsis. Science 329, 1065–1067. 20798316 10.1126/science.1191132

[CIT0062] McAdam SA Brodribb TJ Ross JJ 2016 Shoot-derived abscisic acid promotes root growth. Plant, Cell and Environment 39, 652–659. 10.1111/pce.12669 26514625

[CIT0063] Medina-Rivera A Defrance M Sand O 2015 RSAT 2015: Regulatory Sequence Analysis Tools. Nucleic Acids Research 43, W50–W56. 25904632 10.1093/nar/gkv362 PMC4489296

[CIT0064] Meng L Buchanan BB Feldman LJ Luan S 2012 CLE-like (CLEL) peptides control the pattern of root growth and lateral root development in Arabidopsis. Proceedings of the National Academy of Sciences, USA 109, 1760–1765. 10.1073/pnas.1119864109 PMC3277184 22307643

[CIT0065] Mingossi FB Matos JL Rizzato AP Medeiros AH Falco MC Silva-Filho MC Moura DS 2010 SacRALF1, a peptide signal from the grass sugarcane (Saccharum spp.), is potentially involved in the regulation of tissue expansion. Plant Molecular Biology 73, 271–281. 20148351 10.1007/s11103-010-9613-8

[CIT0066] Mlodzinska E Klobus G Christensen MD Fuglsang AT 2015 The plasma membrane H(+)-ATPase AHA2 contributes to the root architecture in response to different nitrogen supply. Physiologia Plantarum 154, 270–282. 25382626 10.1111/ppl.12305

[CIT0067] Morato do Canto A Ceciliato PH Ribeiro B Ortiz Morea FA Franco Garcia AA Silva-Filho MC Moura DS 2014 Biological activity of nine recombinant AtRALF peptides: implications for their perception and function in Arabidopsis. Plant Physiology and Biochemistry 75, 45–54. 24368323 10.1016/j.plaphy.2013.12.005

[CIT0068] Murphy E De Smet I 2014 Understanding the RALF family: a tale of many species. Trends in Plant Science 19, 664–671. 24999241 10.1016/j.tplants.2014.06.005

[CIT0069] Murphy E Smith S De Smet I 2012 Small signaling peptides in Arabidopsis development: how cells communicate over a short distance. The Plant Cell 24, 3198–3217. 22932676 10.1105/tpc.112.099010 PMC3462626

[CIT0070] Negi S Ivanchenko MG Muday GK 2008 Ethylene regulates lateral root formation and auxin transport in Arabidopsis thaliana. The Plant Journal 55, 175–187. 18363780 10.1111/j.1365-313X.2008.03495.x PMC2635504

[CIT0071] Negi S Sukumar P Liu X Cohen JD Muday GK 2010 Genetic dissection of the role of ethylene in regulating auxin-dependent lateral and adventitious root formation in tomato. The Plant Journal 61, 3–15. 19793078 10.1111/j.1365-313X.2009.04027.x

[CIT0072] Oeller PW Theologis A 1995 Induction kinetics of the nuclear proteins encoded by the early indoleacetic acid-inducible genes, PS-IAA4/5 and PS-IAA6, in pea (Pisum sativum L.). The Plant Journal 7, 37–48. 7894510 10.1046/j.1365-313x.1995.07010037.x

[CIT0073] Ohme-Takagi M Shinshi H 1995 Ethylene-inducible DNA binding proteins that interact with an ethylene-responsive element. The Plant Cell 7, 173–182. 7756828 10.1105/tpc.7.2.173 PMC160773

[CIT0074] Okushima Y Fukaki H Onoda M Theologis A Tasaka M 2007 ARF7 and ARF19 regulate lateral root formation via direct activation of LBD/ASL genes in Arabidopsis. The Plant Cell 19, 118–130. 17259263 10.1105/tpc.106.047761 PMC1820965

[CIT0075] Okushima Y Overvoorde PJ Arima K 2005 Functional genomic analysis of the AUXIN RESPONSE FACTOR gene family members in Arabidopsis thaliana: unique and overlapping functions of ARF7 and ARF19. The Plant Cell 17, 444–463. 15659631 10.1105/tpc.104.028316 PMC548818

[CIT0076] Olsen AN Mundy J Skriver K 2002 Peptomics, identification of novel cationic Arabidopsis peptides with conserved sequence motifs. In Silico Biology 2, 441–451. 12611624

[CIT0077] Parinov S Sevugan M Ye D Yang WC Kumaran M Sundaresan V 1999 Analysis of flanking sequences from dissociation insertion lines: a database for reverse genetics in Arabidopsis. The Plant Cell 11, 2263–2270. 10590156 10.1105/tpc.11.12.2263 PMC144131

[CIT0078] Patharkar OR Walker JC 2015 Floral organ abscission is regulated by a positive feedback loop. Proceedings of the National Academy of Sciences, USA 112, 2906–2911. 10.1073/pnas.1423595112 PMC4352813 25730871

[CIT0079] Pearce G Moura DS Stratmann J Ryan CA Jr 2001 RALF, a 5-kDa ubiquitous polypeptide in plants, arrests root growth and development. Proceedings of the National Academy of Sciences, USA 98, 12843–12847. 10.1073/pnas.201416998 PMC60141 11675511

[CIT0080] Péret B Li G Zhao J 2012 Auxin regulates aquaporin function to facilitate lateral root emergence. Nature Cell Biology 14, 991–998. 22983115 10.1038/ncb2573

[CIT0081] Perry SE Lehti MD Fernandez DE 1999 The MADS-domain protein AGAMOUS-like 15 accumulates in embryonic tissues with diverse origins. Plant Physiology 120, 121–130. 10318690 10.1104/pp.120.1.121 PMC59244

[CIT0082] Petersen TN Brunak S von Heijne G Nielsen H 2011 SignalP 4.0: discriminating signal peptides from transmembrane regions. Nature Methods 8, 785–786. 21959131 10.1038/nmeth.1701

[CIT0083] Reed RC Brady SR Muday GK 1998 Inhibition of auxin movement from the shoot into the root inhibits lateral root development in Arabidopsis. Plant Physiology 118, 1369–1378. 9847111 10.1104/pp.118.4.1369 PMC34753

[CIT0084] Richter GL Monshausen GB Krol A Gilroy S 2009 Mechanical stimuli modulate lateral root organogenesis. Plant Physiology 151, 1855–1866. 19794120 10.1104/pp.109.142448 PMC2785988

[CIT0085] Roberts I Smith S Stes E 2016 CEP5 and XIP1/CEPR1 regulate lateral root initiation in Arabidopsis. Journal of Experimental Botany (in press). 10.1093/jxb/erw231 PMC4983111 27296247

[CIT0086] Scheres B Laskowski M 2016 Root patterning: it takes two to tangle. Journal of Experimental Botany 67, 1201–1203. 26912906 10.1093/jxb/erw049 PMC4762393

[CIT0087] Schwechheimer C Smith C Bevan MW 1998 The activities of acidic and glutamine-rich transcriptional activation domains in plant cells: design of modular transcription factors for high-level expression. Plant Molecular Biology 36, 195–204. 9484432 10.1023/a:1005990321918

[CIT0088] Slane D Kong J Berendzen KW 2014 Cell type-specific transcriptome analysis in the early Arabidopsis thaliana embryo. Development 141, 4831–4840. 25411212 10.1242/dev.116459

[CIT0089] Srivastava R Liu JX Guo H Yin Y Howell SH 2009 Regulation and processing of a plant peptide hormone, AtRALF23, in Arabidopsis. The Plant Journal 59, 930–939. 19473327 10.1111/j.1365-313X.2009.03926.x

[CIT0090] Tang W Perry SE 2003 Binding site selection for the plant MADS domain protein AGL15: an in vitro and in vivo study. Journal of Biological Chemistry 278, 28154–28159. 12743119 10.1074/jbc.M212976200

[CIT0091] Tatematsu K Kumagai S Muto H Sato A Watahiki MK Harper RM Liscum E Yamamoto KT 2004 MASSUGU2 encodes Aux/IAA19, an auxin-regulated protein that functions together with the transcriptional activator NPH4/ARF7 to regulate differential growth responses of hypocotyl and formation of lateral roots in Arabidopsis thaliana. The Plant Cell 16, 379–393. 14729917 10.1105/tpc.018630 PMC341911

[CIT0092] Tavormina P De Coninck B Nikonorova N De Smet I Cammue BP 2015 The plant peptidome: an expanding repertoire of structural features and biological functions. The Plant Cell 27, 2095–2118. 26276833 10.1105/tpc.15.00440 PMC4568509

[CIT0093] Thieme CJ Rojas-Triana M Stecyk E 2015 Endogenous Arabidopsis messenger RNAs transported to distant tissues. Nature Plants 1, 15025. 27247031 10.1038/nplants.2015.25

[CIT0094] Vanden Bossche R Demedts B Vanderhaeghen R Goossens A 2013 Transient expression assays in tobacco protoplasts. Methods in Molecular Biology 1011, 227–239. 23616000 10.1007/978-1-62703-414-2_18

[CIT0095] Van Norman JM Zhang J Cazzonelli CI Pogson BJ Harrison PJ Bugg TD Chan KX Thompson AJ Benfey PN 2014 Periodic root branching in Arabidopsis requires synthesis of an uncharacterized carotenoid derivative. Proceedings of the National Academy of Sciences, USA 111, E1300–E1309. 10.1073/pnas.1403016111 PMC3977299 24639533

[CIT0096] Vermeer JE Geldner N 2015 Lateral root initiation in Arabidopsis thaliana: a force awakens. F1000Prime Reports 7, 32. 25926983 10.12703/P7-32 PMC4371239

[CIT0097] von Wangenheim D Fangerau J Schmitz A Smith RS Leitte H Stelzer EH Maizel A 2016 Rules and self-organizing properties of post-embryonic plant organ cell division patterns. Current Biology 26, 439–449. 26832441 10.1016/j.cub.2015.12.047

[CIT0098] Voβ U Wilson MH Kenobi K 2015 The circadian clock rephases during lateral root organ initiation in Arabidopsis thaliana. Nature Communications 6, 7641. 10.1038/ncomms8641 PMC4506504 26144255

[CIT0099] Wang H Caruso LV Downie AB Perry SE 2004 The embryo MADS domain protein AGAMOUS-Like 15 directly regulates expression of a gene encoding an enzyme involved in gibberellin metabolism. The Plant Cell 16, 1206–1219. 15084721 10.1105/tpc.021261 PMC423210

[CIT0100] Wang H Tang W Zhu C Perry SE 2002 A chromatin immunoprecipitation (ChIP) approach to isolate genes regulated by AGL15, a MADS domain protein that preferentially accumulates in embryos. The Plant Journal 32, 831–843. 12472697 10.1046/j.1365-313x.2002.01455.x

[CIT0101] Weirauch MT Yang A Albu M 2014 Determination and inference of eukaryotic transcription factor sequence specificity. Cell 158, 1431–1443. 25215497 10.1016/j.cell.2014.08.009 PMC4163041

[CIT0102] Whitford R Fernandez A Tejos R 2012 GOLVEN secretory peptides regulate auxin carrier turnover during plant gravitropic responses. Developmental Cell 22, 678–685. 22421050 10.1016/j.devcel.2012.02.002

[CIT0103] Winter D Vinegar B Nahal H Ammar R Wilson GV Provart NJ 2007 An ‘Electronic Fluorescent Pictograph’ browser for exploring and analyzing large-scale biological data sets. PLoS One 2, e718. 17684564 10.1371/journal.pone.0000718 PMC1934936

[CIT0104] Yang F Song Y Yang H Liu Z Zhu G Yang Y 2014 An auxin-responsive endogenous peptide regulates root development in Arabidopsis. Journal of Integrative Plant Biology 56, 635–647. 24479837 10.1111/jipb.12178

[CIT0105] Zheng Y Ren N Wang H Stromberg AJ Perry SE 2009 Global identification of targets of the Arabidopsis MADS domain protein AGAMOUS-Like15. The Plant Cell 21, 2563–2577. 19767455 10.1105/tpc.109.068890 PMC2768919

[CIT0106] Zimmermann P Hennig L Gruissem W 2005 Gene-expression analysis and network discovery using Genevestigator. Trends in Plant Science 10, 407–409. 16081312 10.1016/j.tplants.2005.07.003

[CIT0107] Zimmermann P Hirsch-Hoffmann M Hennig L Gruissem W 2004 GENEVESTIGATOR. Arabidopsis microarray database and analysis toolbox. Plant Physiology 136, 2621–2632. 15375207 10.1104/pp.104.046367 PMC523327

