# Supplementary material for: RALFL34 regulates formative cell divisions in Arabidopsis pericycle during lateral root initiation
Source: J Exp Bot. 2016 Jul 18;67(16):4863–75. doi: 10.1093/jxb/erw281 (PMC4983113; doi:10.1093/jxb/erw281)
Supplement: Supplementary Data [file supp_67_16_4863__index.html]

RALFL34 regulates formative cell divisions in Arabidopsis pericycle during lateral root initiation — RALFL34 regulates formative cell divisions in Arabidopsis pericycle during lateral root initiation — Supplementary Data 

# RALFL34 regulates formative cell divisions in Arabidopsis pericycle during lateral root initiation

## Supplementary Data

Data files

- supplementary\_figures\_S1\_S4.pdf - Supplementary Data
- supplementary\_table\_S1.xlsx - Supplementary Data
