# Supplementary material for: RALFL34 regulates formative cell divisions in Arabidopsis pericycle during lateral root initiation
Source: J Exp Bot. 2016 Jul 18;67(16):4863–75. doi: 10.1093/jxb/erw281 (PMC4983113; doi:10.1093/jxb/erw281)
Supplement: Supplementary Data [file supp_erw281_supplementary_figures_S1_S4.pdf]

RALFL34 regulates formative cell divisions in *Arabidopsis* pericycle during lateral root initiation

Evan Murphy, Lam Dai Vu, Lisa Van den Broeck, Zhefeng Lin, Priya Ramakrishna, Brigitte Van De Cotte, Allison Gaudinier, Tatsuaki Goh, Daniel Slane, Tom Beeckman, Dirk Inzé, Siobhan M. Brady, Hidehiro Fukaki, and Ive De Smet

SUPPLEMENTARY INFORMATION

Supplemental Figures

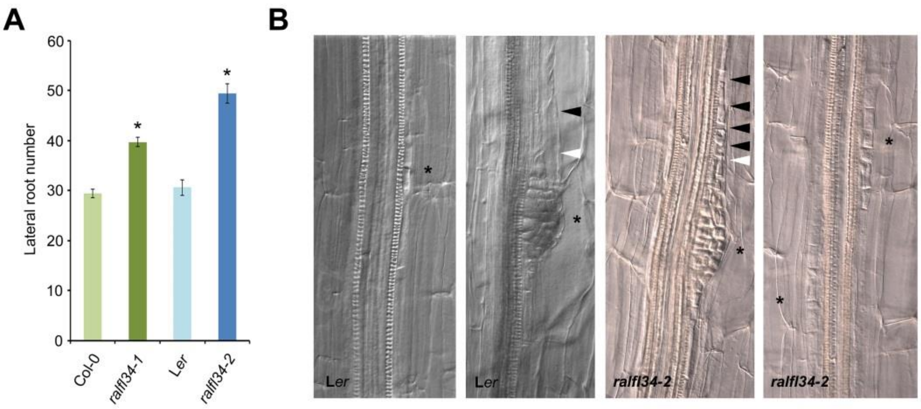

Figure S1. (A) Lateral root number in *ralfl34-1* (n = 69) and *ralfl34-2* (n = 29) compared to their respective controls, Col-0 (n = 79) and L*er* (n = 20) depicted as total root number, including all stages. Graph shows average ± standard error of indicated sample numbers. *, $p < 0.05$ according to Student's *t*-test compared to control. **(B)** Representative DIC pictures of aberrant lateral root development or positioning in *ralfl34-2* compared to control, L*er*. Asterisk indicates lateral root primordium. Arrowheads flank of lateral root primordium (white) and extra rounds of divisions (black).

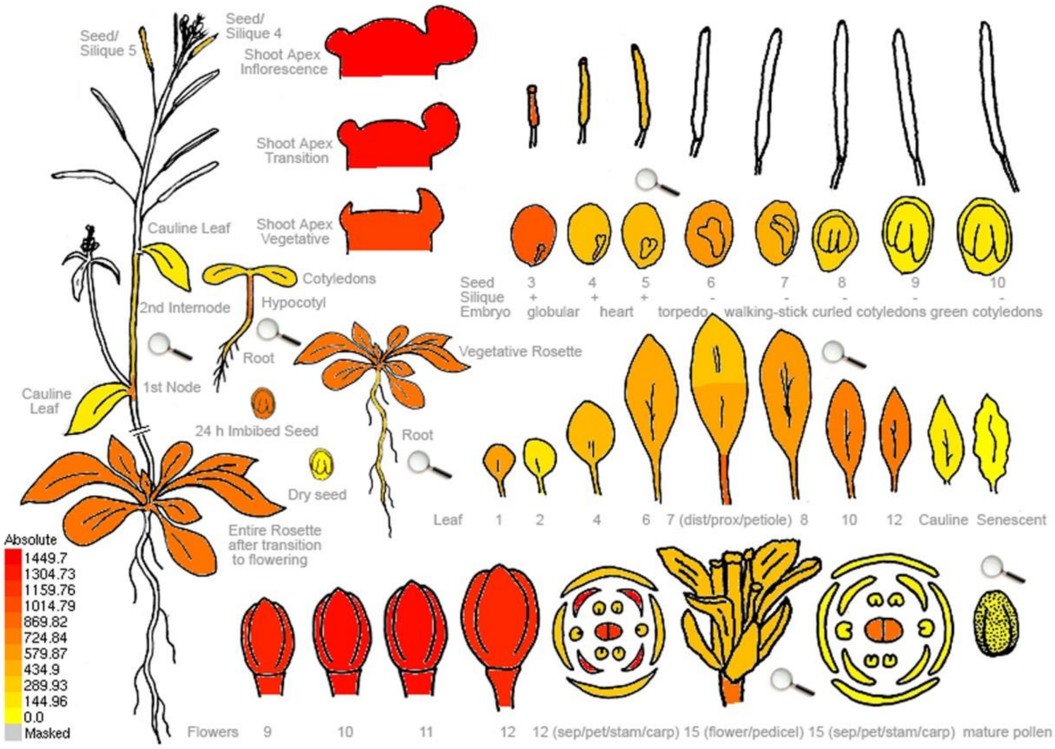

Figure S2. Absolute expression value for *RALFL34* in above ground organs (taken from Arabidopsis eFP Browser).

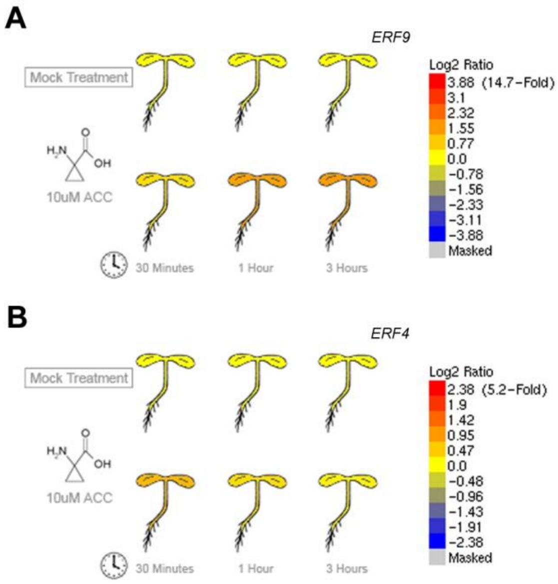

Figure S3. Relative expression values for *ERF4* and *ERF9* upon ACC treatment (taken from Arabidopsis eFP Browser).

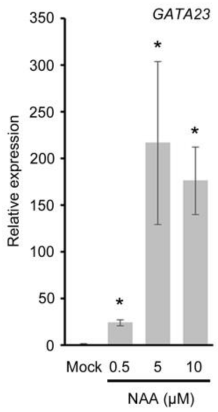

Figure S4. *GATA23* expression upon 6 hours NAA treatment at indicated concentrations. Graph shows average ± standard error of 3 biological repeats. *, $p < 0.05$ according to Student's *t*-test compared to mock.
